# Complete Genome Sequence of Burkholderia gladioli Myophage Mana

**DOI:** 10.1128/MRA.00402-21

**Published:** 2021-05-20

**Authors:** Brenda Godoy, Guichun Yao, Tram Le, Maria Guadalupe Vizoso-Pinto, Jason Gill, Carlos Gonzalez, Mei Liu

**Affiliations:** a Center for Phage Technology, Texas A&M University, College Station, Texas, USA; b Infection Biology Lab, Instituto Superior de Investigaciones Biológicas, National Council of Scientific and Technological Research, National University of Tucuman, San Miguel de Tucumán, Argentina; DOE Joint Genome Institute

## Abstract

Burkholderia gladioli is known to cause respiratory tract infections in cystic fibrosis patients. Here, we describe the annotation of the 38,038-bp genome sequence of Mana, a P2-like phage of *B. gladioli*. Understanding the genomic characteristics of phages infecting pathogens like *B. gladioli* can lead to advancements in phage therapy.

## ANNOUNCEMENT

Burkholderia gladioli is a ubiquitous Gram-negative bacterium (1). Though initially recognized as a plant pathogen, *B. gladioli* has been found to infect the human respiratory tract, predominantly attacking cystic fibrosis patients and other immunocompromised individuals (2). Here, we discuss the genome of *B. gladioli* phage Mana, in an effort to investigate the potential clinical applications of phages to bacterial infections (3).

Bacteriophage Mana was isolated from a soil sample collected from Champaign County, IL, using *B. gladioli* strain ATCC 19302 as the host with the soft agar overlay method, and phage purification was carried out by picking and replating isolated plaques for three rounds on soft agar overlay seeded with the host strain as described previously (4). The host strain was grown at 37°C in tryptic nutrient broth or agar. After phage isolation, phage genomic DNA was extracted from the polyethylene glycol (PEG)-precipitated phage particles and purified using a Wizard DNA cleanup kit as previously described (5), and libraries were prepared with 300-bp inserts using a Swift BioSciences 2S Turbo kit followed by Illumina MiSeq sequencing using v2 300-cycle chemistry. FastQC was used for quality control of the total 519,288 raw sequence reads (www.bioinformatics.babraham.ac.uk/projects/fastqc). The genome sequence was then assembled using SPAdes v3.5.0 (6), to 415.1-fold coverage, and closed using PCR and Sanger sequencing of the product amplified by the primers 5′-CCGACTCGTGGCCTAAA-3′ and 5′-TCTTCACGGATGGACACG-3′. Structural annotation was performed using Glimmer v3 and MetaGeneAnnotator v1.0 to identify the gene sequences, while ARAGORN v2.36 was used to detect tRNAs (7–9). The function of genes was predicted using BLAST v2.9.0 against the NCBI nonredundant (nr) and Swiss-Prot databases, with a maximum E value of 0.001 (10, 11). In addition, InterProScan v5.33 and TMHMM v2.0 were used for functional predictions by conserved domains and transmembrane domains, respectively (12, 13). progressiveMauve v2.4 was used to calculate the genome-wide DNA sequence similarity between Mana and other phages (14). These annotation tools were accessed on the CPT Galaxy and Web Apollo interfaces (15–17), and all analyses were conducted with default settings.

Phage Mana has a genome length of 38,038 bp, a coding density of 94%, and a G+C content of 64%. Structural and functional annotation predicted 64 protein-coding sequences, with 42 of these sequences having an assigned putative function. The Mana genome contains no predicted introns. There were no predicted tRNA-coding sequences. Phage Mana has identifiable P2-like baseplate proteins, a tail tube, a tail sheath, and a tape measure protein, strongly indicating that Mana is a myophage. Mana shows similarity, on both the DNA and protein levels, to previously characterized P2-like *Burkholderia* phages. Among these phages, Mana shares 39 similar proteins with phage vB_BceM_AP3 (GenBank accession no. KP966108) (18) and 34 and 36 similar proteins (BLASTP at an E value of <0.001) with KS5 (GU911303) and KL3 (GU911304), respectively (19). Most of the functions of the Mana genes coincide with the functions of the genes of a P2 phage. Mana was found to have a tape measure protein gene, with a translational frameshift near an upstream chaperone protein gene. Mana was also discovered to contain two predicted holin genes. These genes resemble those of Salmonella phage Epsilon15, a podophage (20). The lysis cassette is completed with a downstream endolysin gene and an o-spanin gene embedded within an i-spanin gene.

### Data availability.

The genome sequence of phage Mana was deposited under GenBank accession no. MT701591.1 and BioSample accession no. SAMN14609638. The BioProject accession number is PRJNA222858, and the SRA accession number is SRR11558334.
